# A model for crystallography engagement with children in early elementary school with activities and book development

**DOI:** 10.1107/S2056989026007607

**Published:** 2026-07-31

**Authors:** Zoe Y. Marr, Justine Wong, S. Chantal E. Stieber

**Affiliations:** a Chemistry & Biochemistry Department California State Polytechnic University,Pomona California 91768 USA; b Department of Chemistry University of Redlands,Redlands California 92373 USA; Harvard University, USA

**Keywords:** crystallography, children, elementary school, outreach

## Abstract

A crystallography educational outreach activity was developed and implemented in the US kindergarten level (K) (5–6 years old), which included writing and illustrating a new children’s book about crystallography titled, *X-ray Crystallography Adventures! Cat & Elephant’s Sugar Crystal Mystery*. The outreach activity included four rotating centers that encompassed hands-on crystallization experiments, observing crystals under microscopes, learning about the crystallography process through coloring, and connecting crystallography to life through reading. Detailed descriptions of the centers and overall activity should enable the ready adoption of this model by others for encouraging young children to engage with and learn about crystallography. >

## Abstract 2

Introducing crystallography at the kindergarten level is discussed through a center-based activity framework and the development of a children’s book for crystallography.

## Introduction

Crystallography is typically considered an advanced experimental technique that is often not taught until graduate school, although in recent years an increasing number of undergraduate programs have introduced X-ray crystallography in the curriculum. An example of a hands-on undergraduate lab that follows the whole crystallography process used table sugar and Epsom salts as examples for students to crystallize, collect data using benchtop instruments, and refine structural models against the collected data to determine the structures (Beauparlant *et al.*, 2023 ▸). Others have reported crystallization and calorimetry of artificial sweeteners (Wouters & Van Meervelt, 2022 ▸). There are several other examples that focus more on structure refinement using previously collected data, because many institutions lack an instrument or time for data collection (Brannon *et al.*, 2020 ▸), engaging groups via site visits or sending samples (Zheng *et al.*, 2025 ▸; Kenfack Tsobnang *et al.*, 2024 ▸), or those that use case studies as examples (Campbell *et al.*, 2016 ▸; Dong & Zheng, 2021 ▸).

In secondary school or high school, there are even fewer examples of crystallography being taught, and crystallography is generally only introduced through outreach activities (Abrahams *et al.*, 2023 ▸). The most extensive example of this was an eight-week after-school program where students learned about crystallography, grew crystals of alkali salts with 4-hy­droxy­benzoic acid, and analyzed data, where some of the structures were new and resulted in publication (Abrahams *et al.*, 2021 ▸, 2022 ▸, 2023 ▸). The XLAB in Göttingen (Germany) also leads an outreach program with local high schools to grow crystals of aspirin, citric acid, or caffeine, followed by a visit to the X-ray facility to collect data (Irmer, 2025 ▸). An example of engagement for primary school learners includes activities using marshmallows and cocktail sticks/toothpicks to learn about mol­ecular structure (Murray *et al.*, 2024 ▸).

Efforts to engage younger students, such as elementary and high school students include large initiatives such as the US Crystal Growing Competition (US Crystal Growing Competition, 2020 ▸; Bettenhausen, 2016 ▸), Bragg your Pattern (Bragg your Pattern, 2026 ▸), a worldwide competition through the IUCr (Van Meervelt, 2014 ▸, 2017 ▸), and the 2024 Transactions Symposium (Bou-Nader *et al.*, 2025 ▸). The US Crystal Growing Competition allows entries from any student up to 12th grade (17–18 years old), and provides kits to households and classrooms to grow alum crystals, which are judged based on the largest size and best quality. Bragg your Pattern has led various initiatives to engage primary school children across Australia and New Zealand with crystallography. One of the current initiatives provides Crystal Explorer Kits to schools with 800 kits available, and they recently led the building of the world’s largest crystal structure model (of a diamond). The 2024 Transactions Symposium at the American Crystallography Association meeting highlighted education and outreach examples particularly through problem solving and experiential learning.

We aimed to develop a curriculum that could be implemented as an outreach activity for early elementary school children, which is described in the following. The curriculum included four center activities: Center 1: Growing borax crystals, Center 2: Microscopic crystal analysis, Center 3: Coloring crystallography, and Center 4: Reading a story about crystallography. A new crystallography children’s book was also written and illustrated to fill the literary gap in crystallography books for children: *X-ray Crystallography Adventures! Cat & Elephant’s Sugar Crystal Mystery* (Stieber & Wong, 2025 ▸).

## Early elementary crystallography outreach design

The overall concept was to develop an outreach activity to engage children by learning about crystals and crystallography for 20–25 participants that could be implemented in an approximately 1.5 h timeframe in a kindergarten classroom. However, the activities described herein could be readily adapted for older children as well. The activity began with a brief introduction to crystallography and the activities (approximately 15 min), followed by four center activities that the children rotate through in smaller groups (approximately 15 min each with extra time for changeover). The activity was implemented once in a classroom of around 25 children. Each center was led by at least one person. Centers 1 and 2 are focused on hands-on laboratory skills, whereas Centers 3 and 4 build up the student’s conceptual knowledge.

### Learning objectives

Learning objectives (LOs) for the activities included learning (1) how to safely conduct experiments, (2) how to grow crystals, (3) how to evaluate crystals using a microscope, (4) how to record data, (5) to recognize that crystals are made up of smaller parts (atoms), and (6) to understand that the X-rays can be used to the determine the three-dimensional structure of mol­ecules (see Table 1 ▸). These learning objectives closely mirror those in the graduate crystallography course taught at Cal Poly Pomona, CHM 5720: Introduction to crystallography, but are simplified to be suitable for elementary school students. Additional learning objectives that align with typical kindergarten learning objectives are the development of broader skills such as fine motor skills, listening, following directions, asking and responding to questions, identifying patterns, and measuring.

**Learning Objective 1: How to safely conduct experiments.** With safety being of the utmost importance when conducting science, teaching students about personal protective equipment (PPE) and enforcing the wearing of gloves and safety glasses was an integral part of the Center 1. This is also reinforced in reading the new children’s book as the characters had to wear PPE before working in the laboratory (Stieber & Wong, 2025 ▸). For Center 1, the students were also reminded that the chemicals were not to be consumed and how to work cleanly and safely.

**Learning Objective 2: How to grow crystals.** One of the simplest methods for growing a crystal is to dissolve the solid of inter­est in hot water and then allow the mixture to cool. The addition of a nucleation site (for example a pipe cleaner or a string) allows crystals to readily grow upon the substrate. Center 1 was dedicated to students preparing supersaturated solutions and constructing their crystallization setup. Another essential aspect of crystal growth is time and patience, which students learn as it is recommended that they wait at least 24 h (or more) for beautiful crystals to form.

**Learning Objective 3: How to evaluate crystals using a microscope.** A tool commonly used among the crystallography community to evaluate crystal quality is a microscope. Ideal crystals have sharp edges, well-defined faces, and are uniform with no visible cracks or defects. Under a microscope, students visually observed crystals and even evaluated the quality of a crystal by determining how well the samples exhibited these characteristics at Center 2. This helped develop their observational skills and pattern recognition.

**Learning Objective 4: How to record data.** The acquisition and recording of data are vital skills for any scientist. In Center 2, the students practiced their ability to accurately represent what they observed as they drew illustrations of the crystals under the microscope. Ultimately, this activity facilitated the development of their observational skills in conjunction with fine motor skills. If the activity is conducted among students of second grade level or above, the center leader(s) can also encourage students to write down their observations.

**Learning Objective 5: Recognizing that crystals are made up of smaller parts (atoms).** One of the fundamental concepts in chemistry is that all matter is composed of atoms and that atoms are very small (too small to observe by eye). This concept is introduced in both Center 3 and Center 4. Ball-and-stick models were consistently used throughout all the activities, with atoms represented as spheres and bonds as sticks. The students saw depictions of the ball-and-stick model in two-dimensional drawings of sucrose and a tangible three-dimensional buckyball. The scale of atoms is demonstrated in the new children’s book as the characters determine that a slice of cake would contain approximately 7 septillion atoms (Stieber & Wong, 2025 ▸).

**Learning Objective 6: Understanding that X-rays can be used to determine the 3D structure of mol­ecules.** Since atoms and the spacing between them are on the angstrom scale (10^−10^ m), X-rays, which can have wavelengths ranging from 10^−9^–10^−11^ m, can be used to determine the atomic arrangement within a crystal. Both Centers 3 and 4 show scientists using X-rays, specifically an X-ray diffractometer, to determine the three-dimensional structure. Diffraction and electron-density maps were briefly explored. Further expansion of diffraction could be conducted with a simple diffraction grating demo with a laser po­inter.

Learning objectives 5 and 6 are more difficult to grasp conceptually, so both Centers 3 and 4 covered these objectives to help reinforce them.

### Materials and supplies

Materials and supplies for a class of 20 children are listed in the following. Center 1 supplies included 20 × 100 mL plastic beakers, 20 child-sized safety glasses, 20 child-sized disposable gloves, 20 standard-sized plastic spoons, 20 wooden stirring sticks, 1 box of borax, boiling water, 1 permanent marker, and 20 plastic sandwich bags. Center 2 supplies included 4 microscopes, 4 pre-made slides with a variety of crystal colors and shapes, 20 microscope handouts (see supporting information), and 4 sets of colored pencils. Center 3 supplies included 20 sets of 5 coloring sheets that were stapled together (Discovering Biology Through Crystallography, 2020 ▸), 5 sets of coloring pencils, and a mol­ecular model of buckyball (although any other mol­ecular model would also work). Center 4 supplies included a children’s crystallography book, for which we wrote *X-ray Crystallography Adventures! Cat & Elephant’s Sugar Crystal Mystery* published by Meitnerium Press (Stieber & Wong, 2025 ▸). However, an alternative can be *Rosalind Franklin* published by Little People, BIG DREAMS (Sánchez Vegara, 2021 ▸), which was used in the first iteration of this activity.

## Activities and discussion

The four center stations that fulfil these learning objectives were Center 1: Growing borax crystals (LO 1,2), Center 2: Microscopic crystal analysis (LO 3,4), Center 3: Coloring crystallography (LO 5,6), and Center 4: Reading a story about crystallography (LO 1,3,5,6).

### Center 1: Growing borax crystals

**Figure 1:**
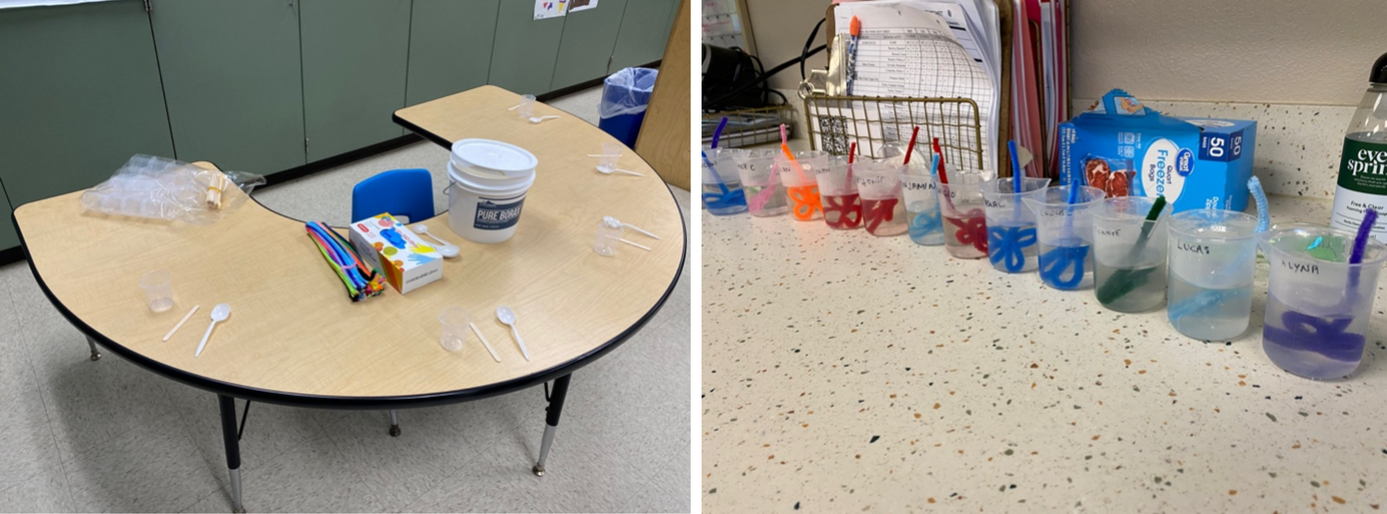
Photograph of Center 1 setup with supplies for borax crystallization (left), and final crystallization setups (right).

The learning objectives for Center 1 were to learn how to safely conduct experiments (LO 1) and how to grow crystals (LO 2). In Center 1 (Fig. 1 ▸), the children set up crystallizations of borax using pipe cleaners, similar to what has been described in many online outreach examples online, but most closely based upon a procedure from Go Science Girls (Go Science Girls, 2019 ▸). All children were provided with child-sized safety glasses and gloves to encourage safe chemistry lab habits. Each child received a 100 mL plastic beaker, safety glasses, and gloves. Instructors helped the children label the glasses and beakers with permanent markers. Children were told that they would grow crystals, but that we were doing real chemistry, and needed to be extra careful. An example of a finished crystallization with borax crystals on a pipe cleaner was shown. First, each child was allowed to choose a pipe cleaner color of their choice and was instructed to bend it into a shape. Then, the instructors helped each child use a plastic spoon to scoop one heaping spoon of borax into their beaker. Finally, the instructors brought the boiling water kettle to the station and filled each beaker with approximately 50 mL of boiling water. The children were reminded to be cautious with the boiling water and were instructed to carefully stir their solutions until they became clear. Finally, the pipe cleaners were placed into the beaker and instructors moved the beaker to a safe place in the classroom for crystallization. The following day, all samples had crystals growing on the pipe cleaners, so teachers transferred the crystallized pipe cleaners to plastic bags for the children to take home.

After this activity, children achieved the learning objectives 1 and 2 based on instructor observations. There were no safety issues, and all children wore the safety goggles and gloves throughout the experiment time. The children were generally very careful and had no spills, and all children produced appropriate crystallization setups. After one day, all of the samples had crystals growing on them. The students had a variety of fine-motor skills, so some needed a bit more help to fashion the pipe cleaner into a shape. The personal protective equipment (PPE) was a novelty for the children, and they were thrilled to take the safety glasses home. It is important to note that names were written on all the safety glasses and beakers, and most children needed help putting on the gloves. Although borax was used in the center activity, other substances such as sugar, sodium chloride, Epsom salt, or potassium alum (used in the US crystal growing competition) would also be suitable for future adaptations of this center.

### Center 2: Microscopic crystal analysis

**Figure 2:**
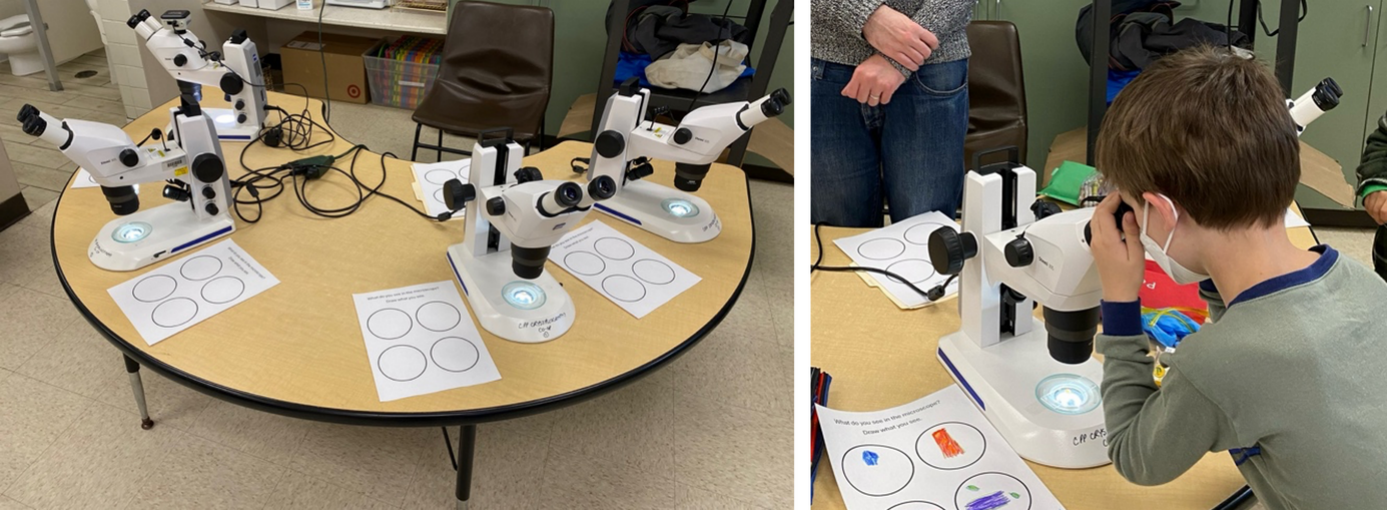
Photo of Center 2 setup for microscope station (left), and child viewing microscope with drawings of crystals (right).

This center (Fig. 2 ▸) focused on students evaluating crystals using a microscope (LO 3), and learning to record their data (LO 4). The station had four microscopes set up, with each already focused on a slide with a unique crystalline sample on it. An effort was made to select crystals that had different colors and shapes. Children were introduced to the microscopes and instructed to be careful because these are very expensive and they are the real research microscopes that scientists use. A worksheet was also dispensed that had the instructions ‘What do you see in the microscope? Draw what you see’ and four empty black circular fields to represent what they might see through a microscope objective (supporting information). Colored pencils were available for students to draw what they saw, and the center leader(s) helped them describe what they saw and what to look for to identify a crystal (sharp edges, even shape, color).

After this activity, children achieved the learning objectives to be able to evaluate crystals using a microscope (LO 3), and learn to record data (LO 4), based on instructor observations. All children were able to draw depictions of what they saw in the microscope and describe the shapes, colors, and whether they saw any sharp edges. The drawings varied a bit because some children chose to be more creative in their artwork, particularly in terms of color choice, and children had different levels of fine motor skills. However, all children participated in recording data by drawing the crystals. Children were particularly impressed with using the microscopes because they were the real microscopes used by scientists at our university. All of the children took good care with the microscopes, including those who are generally more active. The primary note to watch out for is to have an appropriate power strip and extension cord that is out of the way of a walking path, to minimize trip haza­rds.

### Center 3: Coloring crystallography

**Figure 3:**
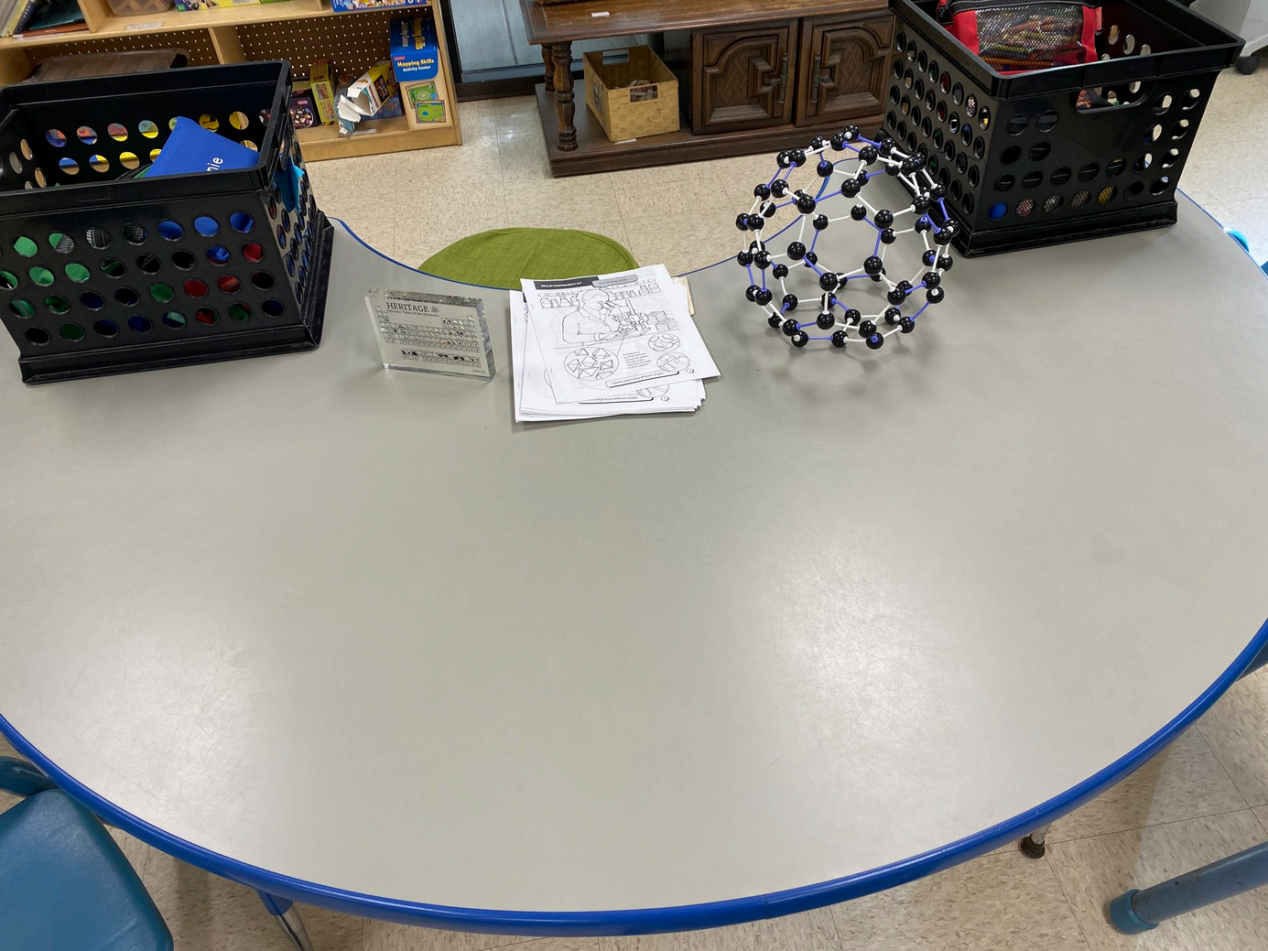
Photo of Center 3 setup with coloring supplies and example buckyball mol­ecular model.

This center (Fig. 3 ▸) focused on learning objectives for children to recognize that crystals are made up of smaller parts (atoms) (LO 5), and to understand that X-rays can be used to determine the 3D structure of mol­ecules (LO 6). Finding coloring pages related to crystallography was surprisingly difficult because many coloring pages available online are for geologic crystals or related to mysticism. However, the Protein Data Bank (PDB) and American Crystallographic Association (ACA) developed a coloring book titled *Discovering Biology through Crystallography*, which is freely available for downloading and includes several pages about the process of crystallography (Discovering Biology Through Crystallography, 2020 ▸). Children were provided with five coloring sheets stapled together developed by the PDB and ACA titled, (*A*) What do crystallographers do?, (*B*) How do crystallographers use the crystals?, (*C*) What happens next?, (*D*) Using crystallography to see small mol­ecules, and (*E*) Understanding larger mol­ecules through crystallography (Discovering Biology Through Crystallography, 2020 ▸). A mol­ecular model of buckyball was also included to aid in the visualization of atomic structure. While the children colored in the sheets, the center leader read the captions on the sheets and explained the process of crystallography including growing crystals (*A*), looking at crystals under a microscope (*A*), collecting data (*B*), processing data (*C*), the atomic structure of sucrose (*D*), and the structure of DNA (*E*).

After this activity, most children achieved the learning objectives that crystals are made up of smaller parts (atoms) (LO 5) and to understand that X-rays can be used to determine the three-dimensional structure of mol­ecules (LO 6), based on instructor observations. Many children had heard of atoms before, and most children had heard of X-rays, but all learned that X-rays could be used to study crystals. All of the participants were completely engaged with the coloring activity, despite the design team being worried about student engagement for this activity. It provided children with a chance to be creative, practice fine motor skills, and also do a more calming activity. Even students who didn’t color all the pages could take the packet home to continue coloring and learning. Future iterations of this activity could add patterns to the coloring sheet for sucrose (*D*) to differentiate the spheres as different atoms, and to put a key on the side so children can tell carbon, oxygen, and hydrogen apart. An expansion of this activity or additional activity that could be implemented would be to offer children model kits to reinforce the concepts of atoms and structure building.

### Center 4: Reading a story about crystallography

This center focused on and reinforced learning objectives of learning how to safely conduct experiments (LO 1), how to evaluate crystals using a microscope (LO 3), to recognize that crystals are made up of smaller parts (atoms) (LO 5), and to understand that X-rays can be used to determine the three-dimensional structure of mol­ecules (LO 6). In the first year this outreach activity was held, the story *Rosalind Franklin* published by Little People, BIG DREAMS was used for the reading station, since it was the only book we could find that discussed crystallography as part of a story. This story is in a biographical form and talks about Rosalind Franklin’s life, inter­est in science, struggles, and achievements and has beautiful illustrations, but it was less focused on the science of crystallography itself. The story was also less engaging and a bit lengthy for the very young kindergarten audience this workshop was designed for, where most children were 5 or 6 years old. In the first iteration of the workshop, the story was summarized using the pictures as a basis for a new, more concise narrative. The limited literature available for young children led our team to conceptualize a new children’s book that highlights the science of crystallography, and will be discussed in the following section.

## Crystallography children’s book development

**Figure 4:**
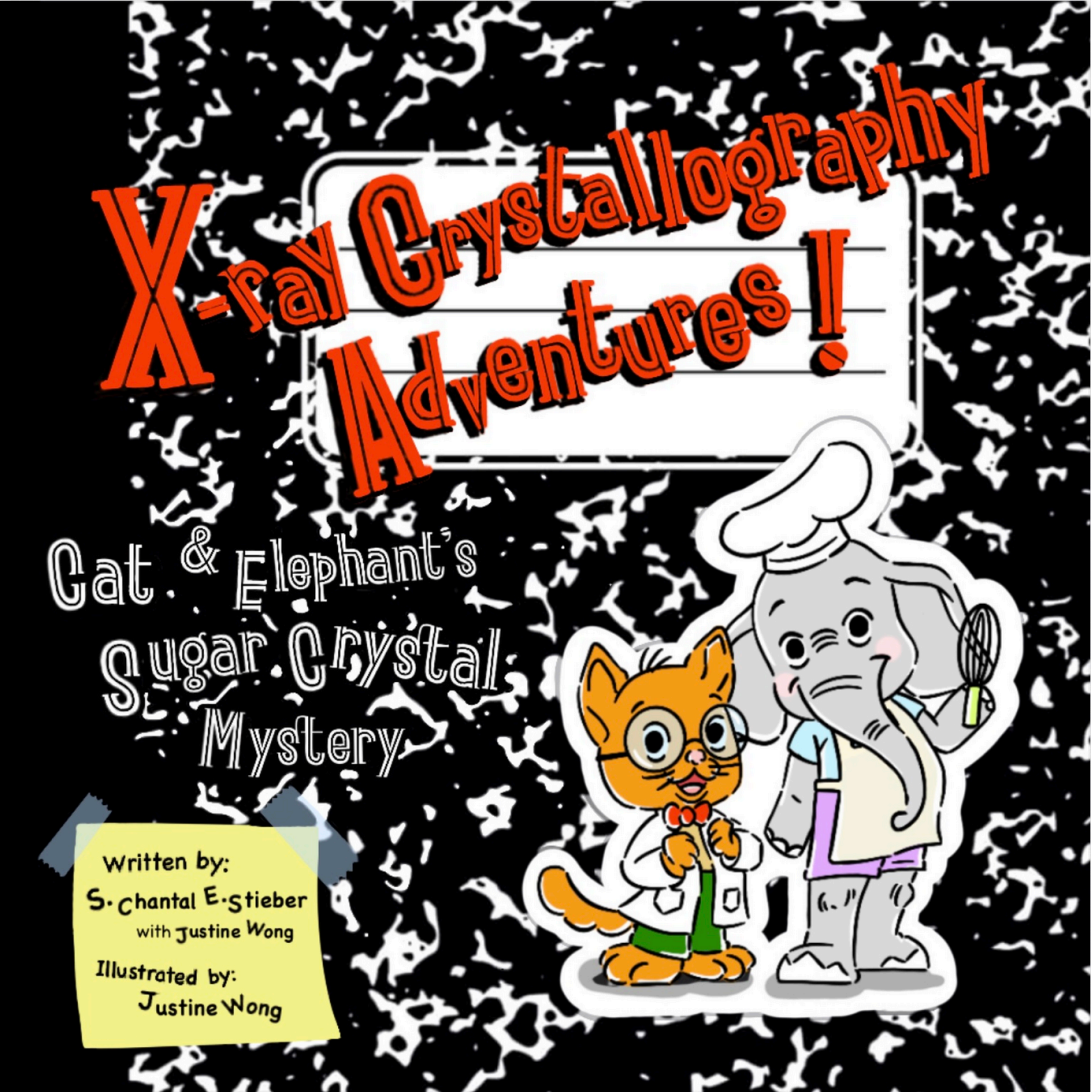
Cover image of *X-ray Crystallography Adventures! Cat & Elephant’s Sugar Crystal Mystery*. Used with permission from Meitnerium Press.

A new children’s book (Fig. 4 ▸) with a story about crystallography was developed titled *X-ray Crystallography Adventures! Cat & Elephant’s Sugar Crystal Mystery* and was published by Meitnerium Press (Stieber & Wong, 2025 ▸). The goal for this story was to relate crystals that scientists study to crystals in everyday life. Little children are often also drawn to shiny things, and thus the idea to relate sugar crystals while baking to crystallography was born. The story follows two characters, Cat who is a chemist, and Elephant who is a baker as they bake a birthday cake for their friend, Mouse. While baking, Elephant wants to know why the sugar is so shiny, and Cat tells Elephant about crystals and that they could put the crystal on the X-ray diffractometer in Cat’s lab to find out the atomic structure. They 3D print the structure, and it is included as part of their present to Mouse. The book features illustrations that are modelled after the Cal Poly Pomona Crystallography Co-op, which houses a Bruker Venture D8 instrument.

The book was written with the following aforementioned learning objectives in mind: how to safely conduct experiments (LO 1), how to evaluate crystals using a microscope (LO 3), to recognize that crystals are made up of smaller parts (atoms) (LO 5), and to understand that the X-rays can be used to the determine the three-dimensional structure of mol­ecules (LO 6). The story includes a page about PPE, two pages about looking into a microscope, several pages about atoms and structure, and a page highlighting shining an X-ray through a sample, followed by diffraction. Feedback for the book has been highly positive, both from crystallographers and non-crystallographers alike. Children of various ages remarked that they appreciated the connection between baking, science, and X-rays. The first book printing included books for donation to schools and for outreach activities. Future iterations of the activity could include bringing in a 3D-printed structure of sucrose, similar to what is introduced in the story. A second crystallography story is currently in development.

## Conclusion

A crystallography outreach activity with four centers was conducted in a kindergarten classroom, and a new children’s crystallography book was developed. The four centers gave students the opportunity to grow borax crystals, view a variety of crystals under a microscope, color images to learn about the process of crystallography, and read a story about baking and crystallography. The activities were relatively easy to implement, achieved the learning objective, and children enjoyed the activities. In particular, many children learned how to grow crystals for the first time and how to use the real microscopes that scientists use in a lab to analyze crystals. The children also engaged with coloring and listening to the story about baking and crystallography to learn more about atoms, using X-rays to study crystals, and the scientific process. This model could be relatively easily implemented in other kindergarten or early elementary school classrooms.

## Supplementary Material

Supporting information file. DOI: 10.1107/S2056989026007607/oi2041sup1.docx

## Figures and Tables

**Table 1:** List of the Center activities and the learning objectives (LO) covered by each

Center Activities	LO 1	LO 2	LO 3	LO 4	LO 5	LO 6
Center 1: Growing borax crystals	×	×				
Center 2: Microscopic crystal analysis			×	×		
Center 3: Coloring crystallography					×	×
Center 4: Reading a story about crystallography	×		×		×	×

## Data Availability

Supplementary information includes details of supplies, handouts and worksheets for the activities.
