# Supplementary material for: A model for crystallography engagement with children in early elementary school with activities and book development
Source: Acta Crystallogr E Crystallogr Commun. 2026 Jul 31;82(Pt 8):998–1003. doi: 10.1107/S2056989026007607 (PMC13446490; doi:10.1107/S2056989026007607)
Supplement: Supplementary file 1 [file e-82-00998-sup1.docx]

Supporting information

The supporting information contains lists of materials and supplies for a class of 20 children (S1-4), handouts or links to handouts (S3, S5), and instructor notes (S6-S9).

S1. Center 1 Supplies:

- 20 x 100 mL plastic beakers
- 20 child-sized safety glasses
- 20 child-sized disposable gloves
- 20 standard-sized plastic spoons
- 20 wooden stirring sticks
- 1 box of borax
- 1 kettle of boiling water
- 20 plastic sandwich bags.
- 1 permanent marker
- 1 finished borax crystallization example

S2. Center 2 Supplies:

- 4 microscopes
- 4 pre-made slides with a variety of crystal colors and shapes
- 20 microscope handouts (see S5)
- 4 sets of coloring pencils

S3. Center 3 supplies:

- 20 sets of 5 coloring sheets available for free online that were stapled together (Discovering Biology Through Crystallography, 2020)
  - Web link: <https://pdb101.rcsb.org/learn/coloring-books/discovering-biology-through-crystallography/>
  - Handout A) What do crystallographers do?
  - Handout B) How do crystallographers use the crystals?
  - Handout C) What happens next?
  - Handout D) Using crystallography to see small molecules
  - Handout E) Understanding larger molecules through crystallography
- 5 sets of colored pencils
- molecular model of buckyball (although any other molecular model would also work).

S4. Center 4 Supplies:

- Children’s crystallography book. We suggest one of the following:
  - “X-ray Crystallography Adventures! Cat & Elephant’s Sugar Crystal Mystery” (Stieber & Wong, 2025).
  - “Rosalind Franklin” (Sánchez Vegara, 2021).

S5. Center 2 Handout: microscopes

S6. Instructor Notes: Center 1

These instructions are most closely based upon a procedure from Go Science Girls (Go Science Girls, 2019).

- Setup the station for 5 children at one time by placing the following items in front of each chair: plastic beaker, stir rod, and spoon.
- When the children arrive at the station, instruct them to sit down.
- Children were told that they would grow crystals, but that we were doing real chemistry, and needed to be extra careful.
- Go over the safety instructions that safety glasses protect their eyes from chemical splashes.
- Give each child a pair of safety glasses and help them write their name on the side with a permanent marker. Instruct them to put on their safety glasses.
- Go over the safety instructions that gloves protect their hands from chemicals.
- Give each child a pair of gloves. Young children will likely need help putting these on.
- An example of a finished crystallization with borax crystals on a pipe cleaner was shown. Children were told that they would be growing crystals using borax, which is often used for cleaning clothes.
- Help each child write their name on the beaker with the permanent marker.
- Allow each child to choose a pipe cleaner color of their choice and instruct them to bend it into any shape they wish that could fit into the beaker. Some children will likely need help with bending the pipe cleaner.
- One by one, ask each child to use a plastic spoon to scoop 1 heaping spoon of borax into their beaker.
- Let the children know that the next step is to add boiling water, which is very hot and could cause burns. Only the instructor will touch the kettle and children should stand back.
- The instructor then fills each beaker with approximately 50 mL of boiling water.
- The children are reminded to be extra careful with the boiling water and instructed to carefully stir their solutions with the stirring stick until they became clear.
- Children are told that the solution becomes clear as the borax dissolves in the solution. The hot water is needed to dissolve the borax.
- Finally, children are instructed to put their pipe cleaners into the beaker. The instructor explains that the pipe cleaner provides a place for the crystals to grow as the solution cools.
- The instructors move the beaker to a safe place in the classroom for crystallization.
- The children are instructed to clean up their workspace by using a paper towel and putting their gloves in the trash.
- The following day, check samples for crystals growing and the instructor transferres the crystallized pipe cleaners to labelled plastic bags for the children to take home.

S7. Instructor Notes: Center 2

- The station should be setup with 4 microscopes that each already are focused on a slide with a unique crystalline sample on it, a handout next to each microscope (S5), and a set of colored pencils next to each microscope. Select crystals that have different colors and shapes.
- Introduce children to the microscopes and instruct them to be extra careful because these are very expensive and they are the real research microscopes that scientists use.
- Discuss how microscopes are used to make crystals look larger by magnifying them.
- Provide general instructions about how to use the microscope by looking through the eyepieces. Instruct children not to touch any other parts of the microscope since they are already focused on the samples.
- Tell children to look into the microscopes and describe what they see. Do they see sharp edges? What is the color? Discuss how crystals typically have sharp edges and uniform shapes.
- Instruct children to draw what they see in the micrsocope in one of the circles on their worksheet.
- Once most children seem to be finished drawing, instruct them to rotate to the different stations.

S8. Instructor Notes: Center 3

- The packets of 5 coloring sheets should be placed on a table with colored pencils. The coloring sheets are available for free online (Discovering Biology Through Crystallography, 2020). Web link: <https://pdb101.rcsb.org/learn/coloring-books/discovering-biology-through-crystallography/>
- This center is mostly self-guided with children coloring in the the coloring pages. During coloring, the instructor should read the descriptions out loud on whichever page a given student is working on and answer any questions.
  - Handout A) What do crystallographers do?
  - Handout B) How do crystallographers use the crystals?
  - Handout C) What happens next?
  - Handout D) Using crystallography to see small molecules
    - Here, the instructor should highlight that the balls represent atoms.
  - Handout E) Understanding larger molecules through crystallography

S9. Instructor Notes: Center 4

This center requires no particular special setup, and involves reading a crystallography book out loud to a group of children. The instructor should answer any questions the children have as they arise.

S10. References

Discovering Biology Through Crystallography (2020). Protein Data Bank 2026. <https://pdb101.rcsb.org/learn/coloring-books/discovering-biology-through-crystallography/> (accessed 2026-06)

Go Science Girls. (2019). *DIY Borax Crystal Flowers.* Go Science Girls: Empowering Girls in STEM. <https://gosciencegirls.com/borax-crystal-flowers/> (accessed 2026-06)

Sánchez Vegara, M. I. (2021). *Little People, BIG DREAMS: Rosalind Franklin.* Alba Editorial.

Stieber, S. C. E. & Wong, J. (2025). *X-ray Crystallography Adventures! Cat & Elephant’s Sugar Crystal Mystery*. Meitnerium Press.
